# Memory and superposition in a superspin glass

**DOI:** 10.1038/s41598-021-87345-1

**Published:** 2021-04-08

**Authors:** D. Peddis, K. N. Trohidou, M. Vasilakaki, G. Margaris, M. Bellusci, F. Varsano, M. Hudl, N. Yaacoub, D. Fiorani, P. Nordblad, R. Mathieu

**Affiliations:** 1 Istituto di Struttura della Materia-CNR, 00015 Monterotondo Scalo (RM), Italy; 2 Department of Materials Science and Engineering, Uppsala University, Box 35, 751 03 Uppsala, Sweden; 3 Institute of Nanoscience and Nanotechnology, NCSR “Demokritos”, 153 10 Aghia Paraskevi, Attiki, Greece; 4 Department of Materials and Processes, ENEA, 00123 Rome, CR Casaccia Italy; 5 Department of Physics, Stockholm University, 106 91, Stockholm, Sweden; 6 Institut des Molécules et Matériaux du Mans, UMR CNRS 6283, Le Mans Université, 72085 Le Mans Cedex 9, France; 7 Dipartimento di Chimica e Chimica Industriale, Università di Genova, Via Dodecaneso 31, 16146 Genova, Italy

**Keywords:** Magnetic properties and materials, Nanoparticles

## Abstract

The non-equilibrium dynamics of the superspin glass state of a dense assembly of ~ 2 nm MnFe_2_O_4_ nanoparticles was investigated by means of magnetization, ac susceptibility and Mössbauer spectroscopy measurements and compared to the results of Monte Carlo simulations for a mesoscopic model that includes particles morphology and interparticle interactions. The zero-field cooled (ZFC), thermoremanent (TRM), and isothermal remanent magnetization (IRM) were recorded after specific cooling protocols and compared to those of archetypal spin glasses and their dimensionality. The system is found to display glassy magnetic features. We illustrate in detail, by a number of experiments, the dynamical properties of the low-temperature superspin glass phase. We observe that these glassy features are quite similar to those of atomic spin glasses. Some differences are observed, and interestingly, the non-atomic nature of the superspin glass is also reflected by an observed superspin dimensionality crossover. Monte Carlo simulations—that explicitly take into account core and surface contributions to the magnetic properties of these ultrasmall nanoparticles in direct contact, as well as interparticle interactions—evidence effects of the interplay between (intraparticle) core/surface exchange coupling and (interparticle) dipolar and exchange interactions.

## Introduction

Spin glasses^1,2^ display dynamical properties such as aging, memory, and rejuvenation^3^. Aging reflects a slow equilibration of the spin configuration at a constant temperature in the spin glass phase after a quench from high temperature. Such an aged spin configuration is kept in memory upon further cooling while at the same time new spin configurations are imprinted at shorter lengths scales (rejuvenation)^3,4^. To probe the intrinsic response of the spin glass phase, magnetometry experiments are performed in low magnetic fields, so that a linear response to field changes is achieved and the principle of superposition applies to the magnetic relaxation^4^. Interacting magnetic nanoparticles have been found to display glassy dynamics and undergo (super)spin glass phase transitions, e.g. concentrated ferrofluids^5,6^, dense assemblies of nanoparticles^7–9^ and nanocomposites^10,11^. The magnetic properties of magnetic nanoparticles are affected by the nature and strength of the inter-particle magnetic interaction^11^, magnetic anisotropy^12^, the nanoparticle size and shape distribution^9^, as well as the interplay between inter- and intraparticle interactions stemming from surface^13,14^.

Glassy magnetic states have been reported in MnFe_2_O_4_^15^ nanoparticle systems of various sizes^16,17^. In the current study, we have investigated the superspin glass state of a dense assembly (i.e. particles are in close contact) of ultra-small (diameter 2 nm) MnFe_2_O_4_ nanoparticles. This implies a significant role of the disordered surface affecting both intra (core/surface exchange coupling) and interparticle (dipolar and exchange interactions) effects. We observe that the system undergoes a superspin glass phase transition and we provide evidence, by means of time and temperature dependent memory experiments, that the low field magnetization dynamics is similar to that of conventional (atomic) spin glasses. Monte Carlo simulations, using a mesoscopic model of the nanoparticle system, reproduce the experimental findings, and confirm, accounting for core-surface and interparticle contributions, the significance of the interplay between intra- and interparticle effects**.** The relative contribution of dipolar and exchange (involving the surface shells) interparticle interactions is determined by simulating the memory experiment in absence of one of them. The simulations indicate that both types of interactions contribute to the observed memory effect and that the contribution of dipolar interactions is much stronger than that of exchange ones, which alone would produce a much smaller effect.

## Results and discussion

Earlier studies on the MnFe_2_O_4_ powder^18,19^ showed that the mean crystallite size obtained by XRD analysis is ~ 2 nm, whereas the value obtained by powder specific area (278 m^2^/g) is about 4 nm. This discrepancy suggests that the particles consist of a few aggregated crystallites. Remanence magnetization measurements by means of DCD and IRM protocols recorded at 5 K clearly show the presence of long range dipolar interparticle interactions. Detailed discussion of these results is reported in the Supplementary Materials.

To further investigate the magnetic structure of these small particles, ^57^Fe Mössbauer spectrometry under intense external magnetic field (MSMF) was performed; all the spectra were analyzed using the program Mosfit^20^. MSMF allows a more reliable distinction between Fe ions located in interstitial sites with tetrahedral (A) and octahedral (B) oxygen coordination (the applied field is usually added to the A-site hyperfine field and subtracted from the B-site hyperfine field allowing a smaller overlap between the two components than in zero field Mössbauer spectra)^21,22^. Furthermore, MSMF spectra can also give information about the magnetic structure of the nanoparticles. In the presence of an external magnetic field parallel to the gamma ray direction, the relative areas of the six lines give information about the degree of alignment of the magnetic moments with the applied field.

The ^57^Fe Mössbauer spectrum (Fig. S2) has a complex shape and it is broadened while the intermediate lines show somehow high intensity and the broadening of the B-sites lines is more pronounced than the A-sites lines. A three-component model was necessary to fit the spectrum (see Supplemental Materials for details). MSMF spectrum indicates the presence of a ferrimagnetic (FiM) and an antiferromagnetic (AF) like phase. This result is in agreement with the Rietveld analysis, identifying the two phase as MnFe_2_O_4_ and (Mn_1/3_Fe_2/3_)O_2/3_(OH)_4/3_, probably arranged in a core–shell structure^19^. MnFe_2_O_4_ has ferrimagnetic structure, whilst a high frustration due to a reduced symmetry^23^ induces an AF-like behavior.

**Figure 1:**
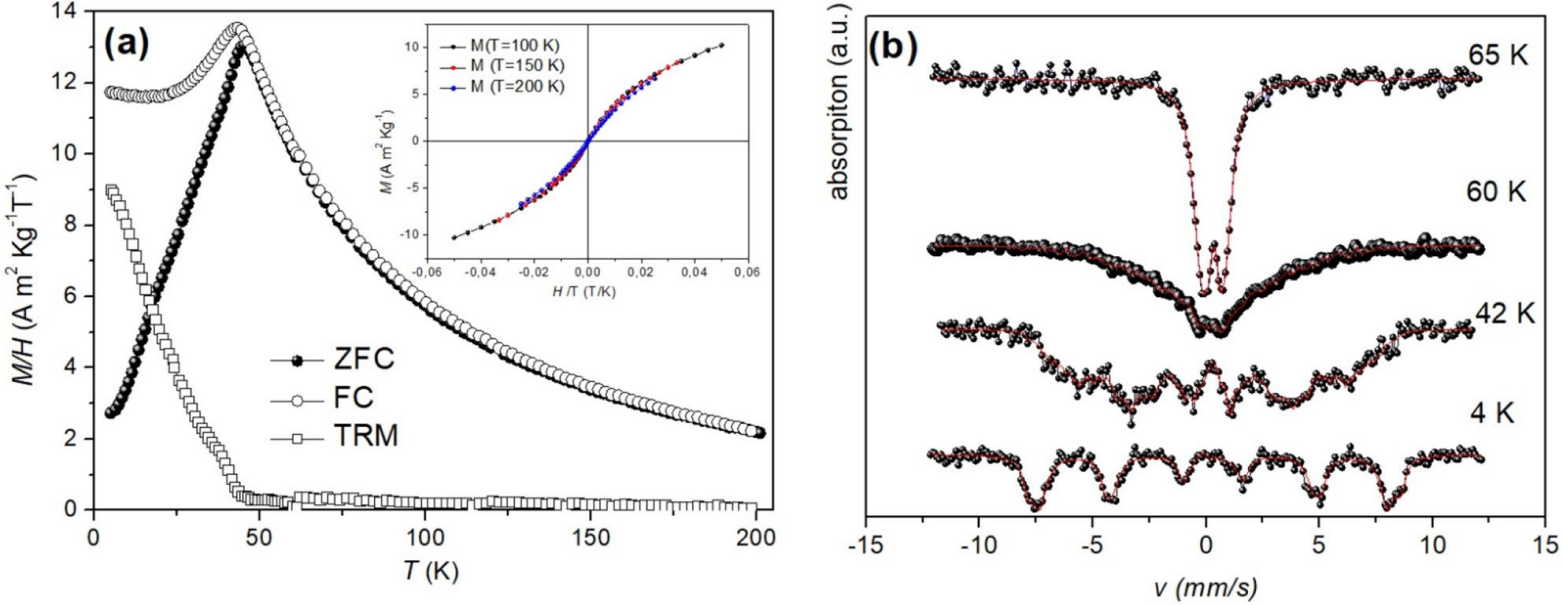
(**a**) ZFC (full circles), FC (empty circles) and TRM (empty squares) curves recorded in H = 10 Oe; inset M Vs (H/T) curves recorded in the range 100 – 200 K; (**b**) ^57^Fe Mössbauer spectra of MnFe_2_O_4_ samples recorded in the range 4–65 K.

In order to investigate magnetization dynamics of the nanoparticles, AC susceptibility and DC magnetization measurements and zero field Mössbauer spectrometry at different temperature have been performed. Figure 1a shows ZFC /FC/ TRM magnetization curves (see “Methods” and Supplemental Materials for a description of these experiments). The FC and ZFC curves coalesce at temperatures just above the maximum in the ZFC, indicating a superparamagnetic behavior of the nanoparticles at higher temperatures. This is confirmed by M Vs H curves recorded between 100 and 200 K which collapse on a single curve when plotted as M Vs H/T (inset Fig. 1a), confirming the superparamagnetic behavior of the nano-entities at these high temperatures^24,25^. The superparamagnetic onset above T_max_ is also confirmed by thermoremanent magnetization, which approaches zero at the temperature where FC and ZFC curves merge. M_ZFC_ shows a maximum at T_max_ ~ 45 K; below this temperature M_FC_ shows a maximum (~ 43 K), then becomes temperature independent and finally shows a slight upturn at the lowest temperature. This behavior resembles that of spin glasses^3^ and has been observed in monodisperse systems of strongly interacting magnetic particles^7–9^. As discussed in Supplementary Materials (Sect. 5), the presence of strong magnetic interaction between the particles is confirmed by magnetic-field dependent remanent magnetization measurements by means of DCD and IRM protocols recorded at 5 K.

Figure 1b shows ^57^Fe Mössbauer spectra recorded at different temperatures in the range 4–65 K. Mössbauer spectra of magnetic nanoparticle assemblies typically consist of a superposition of a sextet due to particles with long relaxation time compared to the time scale (⁓ 5 × 10^−9^ s) of Mössbauer spectrometry and a doublet due to particles with shorter relaxation time compared to it. The relative area of the doublet increases with increasing temperature. The blocking/freezing temperature in Mössbauer spectroscopy T_Moss_, may be defined as the temperature at which the spectral areas of the sextet and the doublet are equal. From analyses of our Mössbauer spectra at different temperatures (some spectra are shown in Fig. 1b), T_Moss_ ≈ 62 K has been determined. Mössbauer spectroscopy and DC magnetization measurements have significantly different time scales (⁓ 10–30 s for magnetization measurements), and thus the freezing/blocking temperature T_max_ estimated using the two techniques are expected to differ considerably. In the literature, it is reported that the ratio T_Moss_/T_max_ decreases with increasing interparticle interactions^26,27^. For the blocking temperature of non-interacting particles, obeying Arrhenius dynamics, the ratio T_Moss_/T_max_ is about 6**,** whilst lower values are reported for spin glass and superspin glass systems^28^, where critical dynamics govern the behavior. For our sample the ratio T_Moss_/T_max_ is ⁓ 1.4.

**Figure 2:**
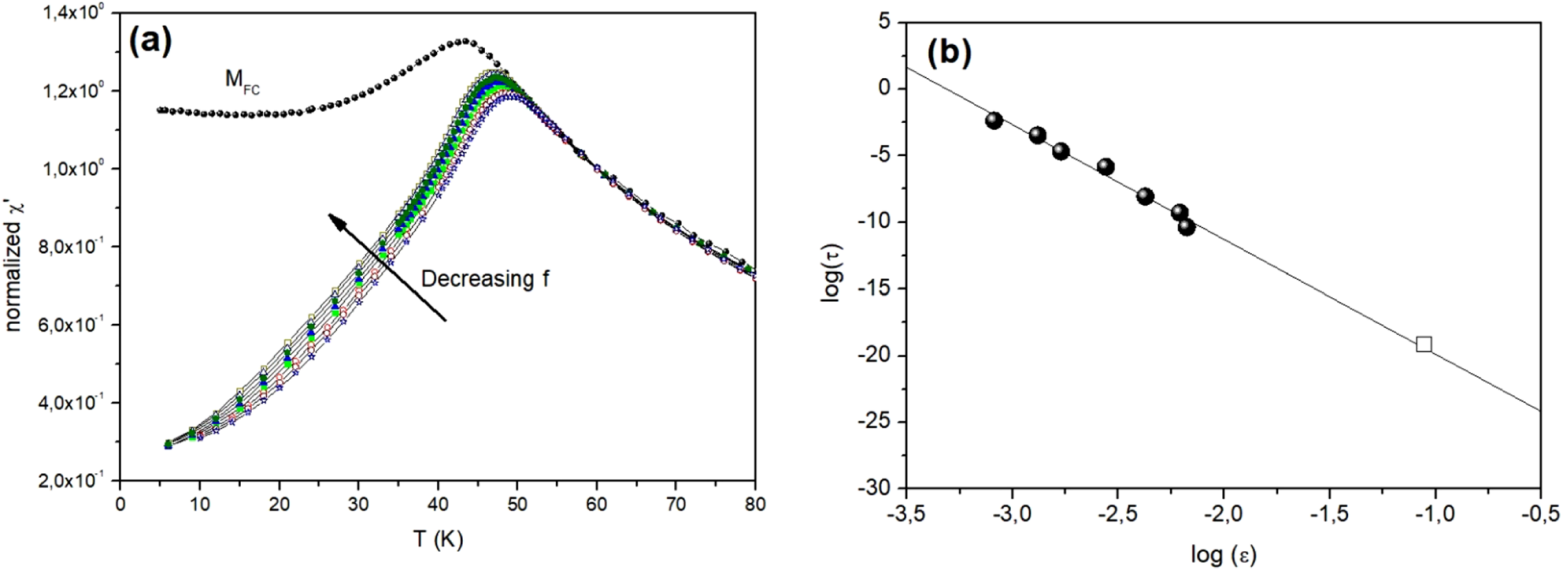
(**a**) Temperature dependence of the in-phase component of the ac-susceptibility recorded for different frequencies; field amplitude *h* = 4 Oe (*f* = 1.7, 5.1, 17, 55, 170 Hz), and *h* = 17 Oe (*f* = 170, 510, 1700, 5100 Hz). The temperature dependence of the field cooled (FC) magnetization collected in H = 10 Oe is added for comparison. All data is normalized by χ' (*f* = 1.7 Hz, *T* = 60 K). (**b**) scaling of the relaxation time τ with the reduced temperature ε = (*T* *−* *T*_*g*_)/*T*_*g*_. Filled markers represent the (T_f_) data obtained from the ac-susceptibility data; open marker is obtained from Mössbauer spectrometry.

The ac-susceptibility was recorded for different frequencies (f) and the in-phase component of the susceptibility χ’(T,f) is shown in Fig. 2a. Considering the FC magnetization data (plotted as M/H) as equilibrium susceptibility, one can see that the various χ’(T,f) curves closely follow the equilibrium curve above 50 K in the superparamagnetic state. Below this temperature, the curves start to deviate. We can define a freezing temperature *T*_*f*_ for each frequency, below which the longest relaxation time (τ) of the system exceeds the observation time of the measurement, τ ~ 1/(2π*f*). (See Fig. S3 in Supplementary Materials). Such a freezing temperature may be defined for each frequency f, yielding (*T*_*f*_* ,f*) datasets which may be analyzed using various scaling laws. Data obtained from Mössbauer spectroscopy experiments (τ = 5 × 10^−9^ s, T_f_ = 62 K) is added using an open marker. In spin glass systems, the critical slowing down implies that the time necessary to reach equilibrium will become longer and longer when approaching the spin glass phase transition T_g_ (glass temperature) and τ diverges at T_g_ according to the power law τ/τ_0_ = ε^−zν^, where ε is the reduced temperature *(T* *−* *T*_*g*_*)/T*_*g*_ and *z*, ν critical exponents^2^. As seen in Fig. 2b, the *T*_*f*_ data follows a power law behavior on a pretty wide frequency window, with physical values of critical exponents (zν = 9(1)) and flipping times, (τ_0_ = 10^−12 (1)^ s) indicating a (super)spin glass transition at T_g_ = 46 (1) K in the system. The error bars on the parameters derived from the scaling analysis are quite large. Yet, the obtained zν value is comparable to those of 9–11 reported for superspin glasses^5,9^, and in the range of those observed for "isotropic" Heisenberg atomic spin glasses (zν ~ 6–8) and "anisotropic" Ising ones (zν ~ 10–12)^29^. The value of τ_o_ determined in our analysis is lower than typical values for superspin glasses^5^, possibly due the very small size and strong magnetic interaction of the particles.

**Figure 3:**
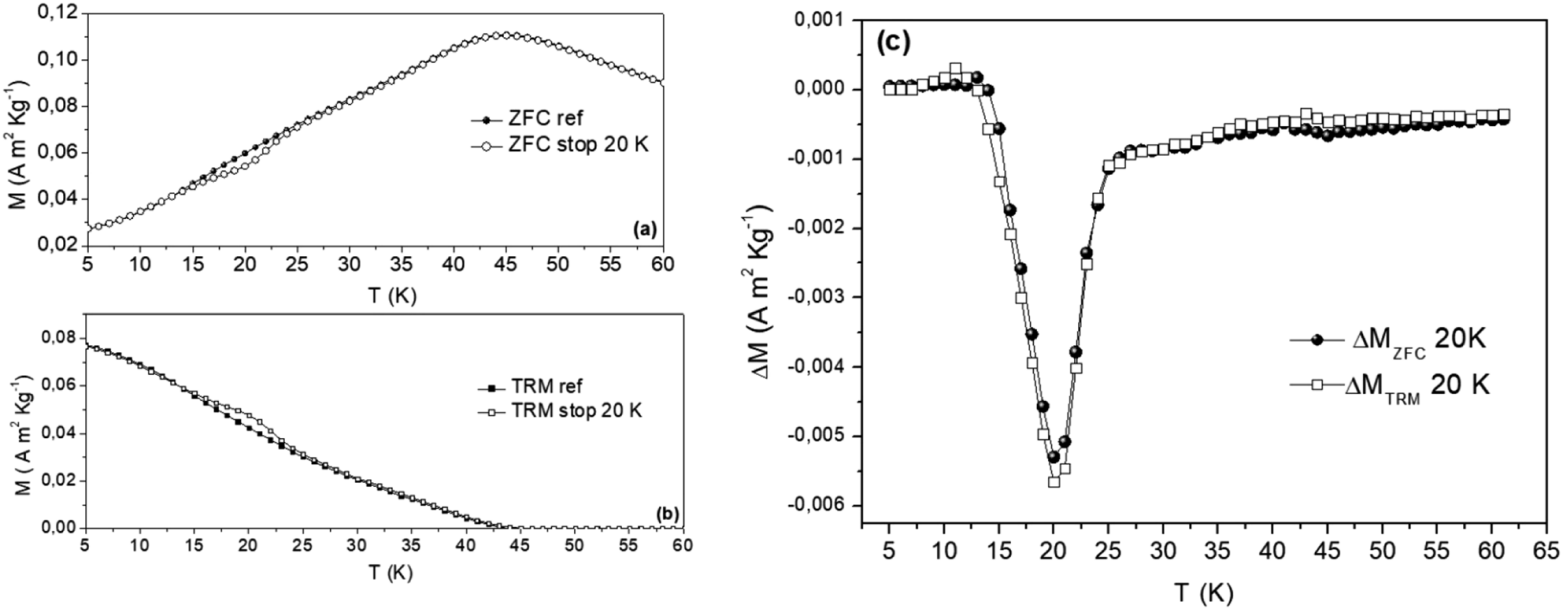
(**a**) ZFC and (**b**) TRM magnetizations for H = 100 Oe. Two curves ZFC and TRM measured after a t_h_ = 10800 s halt at T_h_ = 20 K during cooling are added; (**c**) difference with the corresponding reference curves.

The existence of non-equilibrium dynamics of MnFe_2_O_4_ is investigated by means of time-dependent relaxation (not shown) and temperature-dependent memory experiments performed in small magnetic fields (10 Oe), described in the Supplemental Materials (see Fig. S5 for a sketch of the measurement protocols). Figure 3a,b show the ZFC and TRM reference curves (presented in Fig. 1a), as well as the corresponding curves recorded on reheating after a halt of duration t_h_ = 10800 s at T_h_ = 20 K without magnetic field change (memory curves). In the TRM case the field is thus kept to its H = 10 Oe value, while for the ZFC, it remains zero. The memory curves show a dip (ZFC) or bump (TRM) illustrating the memory and rejuvenation effects. The FC magnetization experiences a minute downward relaxation during such a halt in a constant magnetic field. The principle of superposition connects the response to magnetic field changes and relaxation as M_ZFC_(t_w_,t) = M_FC_(0,t+t_w_) − M_TRM_(t_w_,t)^3^, provided that the field change yields linear response. Using a specific heating rate in ZFC/TRM experiments corresponds to probing the system at a specific observation time (order of 10 s). The similarity of the difference plots between reference and ZFC/TRM memory curves plotted in Fig. 3c hence indicates that the principle of superposition observed in the spin glasses^3^ is valid also for our sample. The excess magnetization at T_h_ in M_TRM_ (T) is equal to the magnetization loss in M_ZFC_ (T) in absolute values.

**Figure 4:**
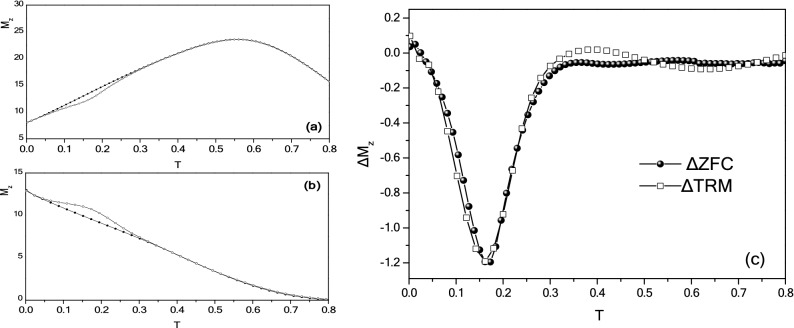
(**a**) Monte Carlo simulations of the ZFC (**a**) and TRM (**b**) magnetizations at H=0.08. Two curves ZFC and TRM calculated after a t_w_ = 5*10^6^ MCSS stop at T = 0.18 while cooling are added; (**c**) difference with the corresponding reference curves.

Memory experiments have been also performed using MC-simulations, considering a mesoscopic model for a dense assembly of ferrimagnetic nanoparticles showing superspin glass characteristics in ZFC-FC curves, as described in Ref^13^. The energy parameters given in the Supplementary Material (equation S1) are based on the bulk values of MnFe_2_O_4_ (M_S_ = 5 × 10^5^ A/m and K = 3 × 10^3^ J/m^3^), and their modifications are established considering the nanoparticles morphology (e.g. reduced symmetry and reduced size) using a mean field approach. The three spin model was employed and the effective intra-particle exchange coupling constants among the core spin and the surface spins were taken as j_c1_ = 0.5, j_c2_ = 0.45, j_srf_ = − 1.0 and the effective anisotropy constants of the core as k_C_ = 0.05 and the surface k_srf_ = 1.0. We take the inter-particle exchange coupling constant as j_inter_ = − 0.50 as a free parameter, the dipolar strength is calculated and found g = 3. Figure 4 shows the Monte Carlo simulations for the reference ZFC and TRM magnetization curves together with the memory curves for t_w_ = 5 × 10^6^ and their difference ΔΜ. The dip of the memory ZFC, the bump of the memory TRM and the similarity of the ΔΜ curves of the two memory experiments confirms that the dynamic properties of the simulated system are characterized by the existence of a superspin glass state. The validity of the model, accounting for interparticle contributions and intraparticle (i.e., intraparticle exchange interaction, namely core/surface and surface exchange interaction, and surface anisotropy), demonstrates that the observed memory effects result from an interplay between interparticle interactions and surface disorder.

**Figure 5:**
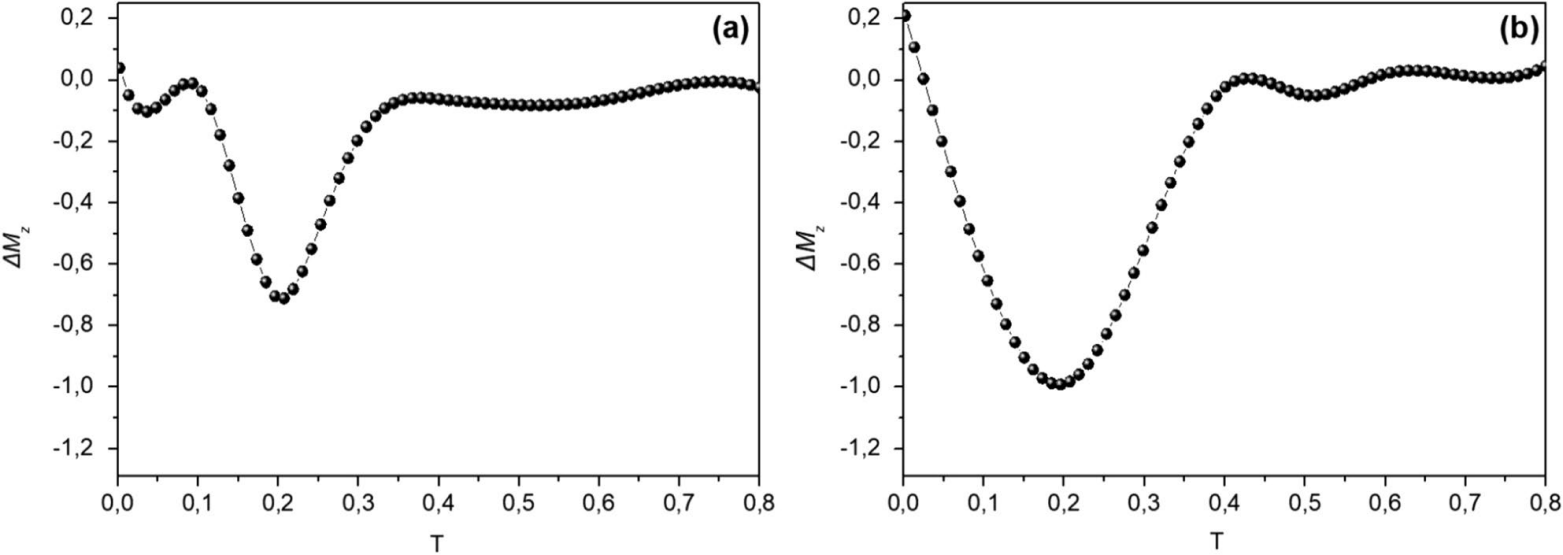
Difference ΔΜ between ZFC reference and ZFC memory curves for: (**a**) the system in absence of dipolar interactions (g = 0); (**b**) the system in the absence of exchange interparticle interactions (j_inter_ = 0).

In order to determine the relative contribution of dipolar and exchange (involving the surface shells) interparticle interactions, we have simulated the memory experiment in absence of one of them, i.e. switching off either j_inter_ or g, respectively. The results (Fig. 5) indicate that both kinds of interactions contribute to the observed memory effect. It is also evident that the contribution of dipolar interactions is much stronger than that of exchange interactions, which alone would produce a much smaller effect (Fig. 5b; absence of dipolar interactions), as we have also pointed out in reference^13^ (see also Supplemental Materials for some insight on intra-particle interaction effects). Weak rejuvenation effects have been reported in many cases for atomic spin glasses in simulations based on the Edwards Andersson model^30^. Similar results have been obtained for Ising models of dipolarly interacting nanoparticles^31^. However the present Heisenberg model takes into account the interparticle exchange interactions and the anisotropy energies of the weakly anisotropic Mn ferrite nanoparticles together with strong surface spin disorder, and hence may display stronger memory and rejuvenation features.

**Figure 6:**
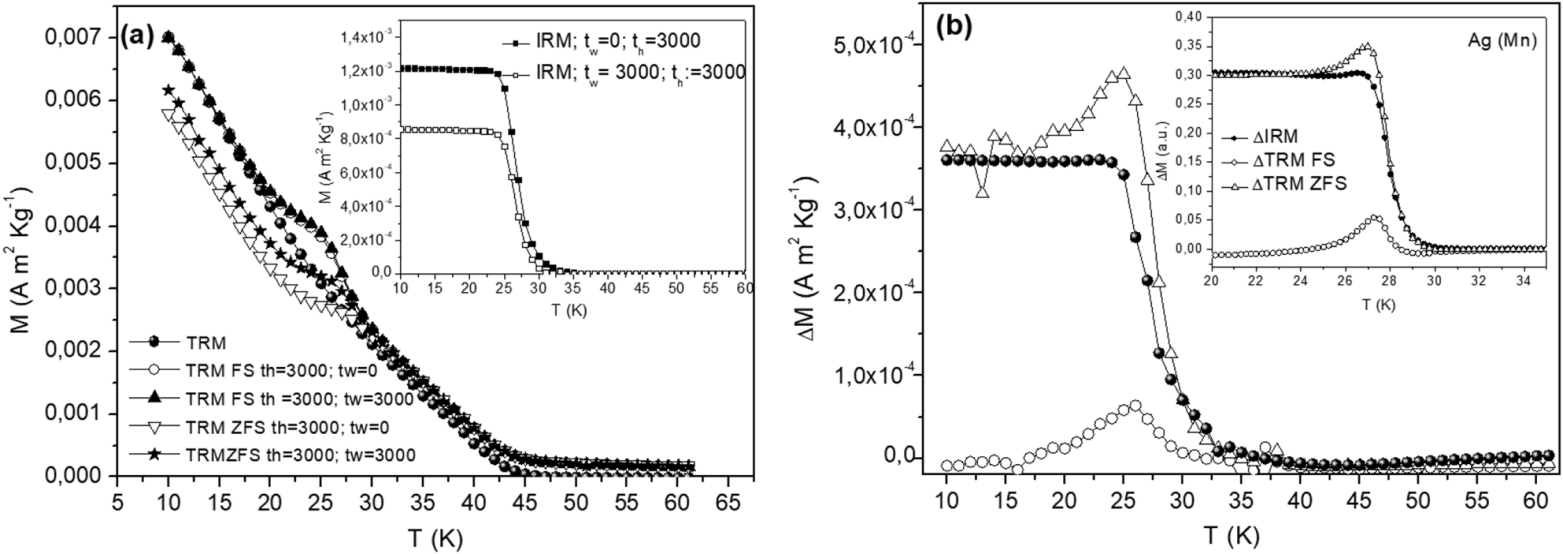
(**a**) TRM magnetization measured after Field Stop (FS) with 10 Oe and Zero Field Stop (ZFS) at 25 K after different waiting (t_w_) and halt time (t_h_) as defined in the text ; (inset **a**) IRM magnetization measured after FS with 10 Oe at 25 K for different t_w_ and t_h_ as defined in the text; (**b**) different between pair of curves reported in (**a**): ΔM_IRM,_ ΔM_TRM_FS (plotted as − ΔM_TRM_FS ) and ΔM_TRM_ZFS (plotted as − ΔM_TRM_ ZFS) for MnFe_2_O_4_ (main frame) and the archetypal spin glass Ag(Mn) (inset).

The memory experiments described above corresponds to field stop (FS) experiments in which the magnetic field is kept to its initial value (zero for ZFC and H for TRM) during the time t_h_. The influence of aging (wait time t_w_ dependence) on the magnetic relaxation can be directly monitored in low field isothermal remanent magnetization vs. temperature experiments (M_IRM_(T)) using a similar temperature protocol as in the memory experiments including a field application after different wait times (t_w_) during the halt^3,32^. (t_w_ is the time the sample has been kept at constant temperature before the field change). The excess magnetization attained during the halt freezes in when the field is cut off and cooling resumes after the halt. The inset of Fig. 6a shows M_IRM_(T) recorded on heating using t_w_ = 0 and t_w_ = 3000 s and in both cases a hold time for the magnetic field of 3000 s. As seen in the figure, the magnitude of M_IRM_ depends on the wait time at T_h_ (= 25 K) before the field application, which reflects a wait time dependence (aging) of the zero field cooled magnetization M_ZFC_(t,t_w_). Similar experiments may be devised, considering a zero-field stop (ZFS) procedure for the TRM, in which the magnetic field is switched to zero during the halt^3^. Sketches of the evolution of the temperature and magnetic field with time in the various protocols are included in supplementary materials (Fig. S5). The results of the ZFS and FS M_TRM_(T,t_w_) measurements using the same wait times and hold times as in the IRM experiments are shown in Fig. 6a (inset).

Both the IRM and TRM data (ZFS and FS conditions), reflect, as expected, the aging phenomenon. Comparing the curves collected without wait time (t_w_ = 0) or including a wait time (t_w_ = 3000 s) at the halt temperature before the field application, one may observe how the IRM curves recorded with t_w_ = 3000 s lie significantly lower than the t_w_ = 0 one^3,32^. The difference curves between the experiments performed with and without wait times, denoted ΔM_TRM_ZFS, ΔM_TRM_FS and ΔM_IRM_ are plotted in Fig. 6b. It was argued in Ref.^3^ that ΔM_TRM_ZFS(T), ΔM_IRM_(T), and ΔM_TRM_FS(T) reflect M_TRM,_ M_ZFC_, and M_FC_ respectively, at an observation time given by the heating rate (the same in all these experiments). The superposition of relaxations M_ZFC_(t_w_,t) = M_FC_(0,t+t_w_) − M_TRM_(t_w_,t) would then imply the relation ΔM_IRM_(T) = ΔM_TRM_FS(T) − ΔM_TRM_ZFS(T). This seems to be satisfied for the excess magnetization of the MnFe_2_O_4_ superspin glass, as we observe that ΔM_TRM_ZFS(T) (plotted as − ΔM_TRM_ZFS(T)) is quite similar to ΔM_IRM_(T), except in the vicinity of T_h_, around which ΔM_TRM_FS(T) is non-zero. As seen in Fig. 6b, the ΔM_TRM_ZFS(T), ΔM_IRM_(T), and ΔM_TRM_FS(T) curves for MnFe_2_O_4_ (main frame) are quite similar to those of the archetypal Ag(Mn) spin glass (inset)^3^.

**Figure 7:**
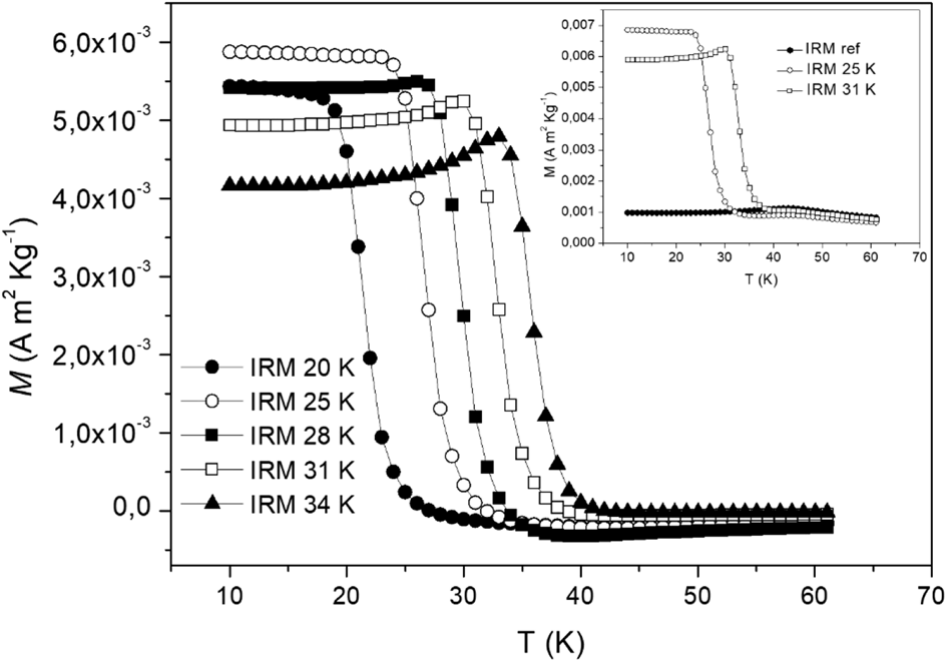
M_IRM_ vs temperature using a perturbation field of 50 Oe and t_h_ = 300 s for several halting temperatures; T_h_ = 20, 25, 28, 31, 34 K. The data was corrected for a background contribution, as illustrated in the inset. The inset shows the magnetization data without correction at T_h_ = 25 K and 31 K as well as the reference background measurement, denoted IRM ref, in which the system is cooled and measured in zero applied magnetic field.

The IRM magnetization curves presented in Fig. 6a for MnFe_2_O_4_ are relatively flat at low temperatures, and decrease above T_h_. This behavior is qualitatively different from the above mentioned Ag(Mn) spin glass, for which an upturn of the magnetization is observed below T_h_^32,33^. In the latter case, the upturn has been related to the spin dimensionality (Heisenberg-like), as Ising spin glasses show IRM curves without upturn, akin to MnFe_2_O_4_^32^. Interestingly, it was observed in Ref.^33^ that the IRM curves of maghemite nanoparticles had different superspin dimensionality, depending on T_h_. The “Heisenberg character” of the IRM curves at temperatures near Tg (with upturn below T_h_) is gradually replaced by an “Ising character” as T_h_ becomes lower and lower. Interestingly a similar crossover is observed in the present MnFe_2_O_4_ system (see Fig. 7) reinforcing, as predicted in Ref.^33^, the idea of an influence of the individual particle relaxation and anisotropy on the apparent superspin dimensionality of the system.

## Conclusions

The dynamical magnetic properties of a superspin glass consisting of a dense assembly of ultra-small MnFe_2_O_4_ nanoparticles have been investigated by means of SQUID magnetometry. The zero-field cooled (ZFC), thermoremanent (TRM), and isothermal remanent magnetization (IRM) were recorded, using specific protocols, to compare the low field magnetization dynamics of that system to that of archetypal spin glasses. Mössbauer spectra were recorded as a function of temperature and magnetic field, yielding information on the dynamical magnetic properties and magnetic structure, respectively. The low-field magnetization dynamics was found similar to that of archetypal spin glasses in spite of the inherent characteristics of nanosystems (superspins instead of atomic spins, surface disorder, interparticle and intraparticle magnetic interaction), reflected in the observed superspin dimensionality crossover. The time and temperature dependence of the ZFC and TRM magnetization were well reproduced by Monte Carlo simulations, using a mesoscopic model of a nanoparticle assembly, with core/shell morphology, accounting for all the intraparticle (core, surface, core/surface interface coupling) and interparticle (dipolar and exchange interactions) effects. This provides an evidence of the interplay between the above effects. The simulation of the memory experiments clearly evidences that the contribution of dipolar interactions is much stronger than that of exchange interactions.

## Methods

### Experimental techniques and data treatment

MnFe_2_O_4_ nano-powders have been synthesized by coprecipitation of Fe^3+^ and Mn^2+^ from water-in-toluene reverse micelle system and subsequent thermal treatment at 320°C. Detailed synthesis procedure, structural and morphological characterization are reported elsewhere^18,19^.

DC magnetization measurements were performed in zero-field-cooled (ZFC), field-cooled (FC), thermo-remanent (TRM) and isothermal remanent (IRM) conditions using a Quantum Design SQUID magnetometer equipped with a superconducting coil (H_max_ = 5 T). The temperature-dependent ZFC, FC, TRM, and IRM magnetization measurements, as well as the magnetic field-dependent direct current demagnetization (DCD) and IRM are described in more details in the Supplemental Materials. To avoid any movement of the nanoparticles during the measurements, the samples, in the form of powders, were immobilized in epoxy resin. The ac-susceptibility χ data was recorded as a function of the temperature T and frequency ω = 2π*f* on the same system as well as on a Quantum Design physical property measurement system (PPMS).

### Monte Carlo model

We have used the Monte Carlo (MC) simulations technique to calculate the memory behavior of the dense assembly of ultra-small Mn ferrite nanoparticles. A detailed description of our mesoscopic model that includes the core/surface morphology of each particle and the interparticle interaction is given in Ref.^13^. Here, in the Supplementary materials we have also included a brief description of this model in order to provide the parameters entering our simulations. In short, three spins (one core and two surface ones) are considered for each particle, yielding exchange interaction (with strengths J_c_ and J_srf_) and anisotropy terms (K_c_ and K_srf_). The interparticle interaction is considered by including exchange (J_inter_) and dipolar (g) interaction. For the calculation of the Reference and Memory ZFC and TRM curves we follow the experimental procedure: (1) the system was first cooled at a constant temperature rate from T = 0.8 in zero field in the ZFC magnetization procedure and by applying a low field H = 0.08 for the calculation of the TRM curves. (2) hold-and-wait at temperature T_h_ = 0.18 for a waiting time (ZFC and TRM) t_w_ = 5 × 10^6^ Monte Carlo Steps per Spin (MCSS). (3) Continue cooling down the system to the lowest temperature T = 0.002 (4) heating the sample in the presence of a magnetic field H = 0.08 for ZFC; for the TRM, the field was removed. The magnetization was monitored in the step (4) for each temperature. Detailed description of the model is reported in Supplementary Materials.

## Supplementary Information

Supplementary Information.
