# Supplementary material for: Memory and superposition in a superspin glass
Source: Sci Rep. 2021 Apr 8;11:7743. doi: 10.1038/s41598-021-87345-1 (PMC8032676; doi:10.1038/s41598-021-87345-1)
Supplement: Supplementary file 1 — Supplementary Information. [file 41598_2021_87345_MOESM1_ESM.pdf]

Supplemental Materials for

Memory and superposition in a superspin glass

*D. Peddis *[1,7], K.N. Trohidou[3], M. Vasilakaki[3], G. Margaris[3], M. Bellusci[4], F. Varsano[4], M. Hudl[5], N. Yaacoub [6]*

D. Fiorani[1], P. Nordblad[2] and R. Mathieu[2]*

1 Istituto di Struttura della Materia-CNR, 00015 Monterotondo Scalo (RM), Italy

2 Department of Materials Science and Engineering, Uppsala University, Box 35, SE-751 03 Uppsala, Sweden

3 Institute of Nanoscience and Nanotechnology, NCSR "Demokritos", 153 10 Aghia Paraskevi, Attiki, Greece

4 Department of Materials and Processes, ENEA, CR Casaccia, 00123 Rome, Italy

5 Department of Physics. Stockholm University, 106 91, Stockholm, Sweden

6 Institut des Molécules et Matériaux du Mans, UMR CNRS 6283, Le Mans Université, 72085 Le Mans Cedex 9, France

7 Dipartimento di Chimica e Chimica Industriale, Università di Genova, Via Dodecaneso 31, I-16146 Genova, Italy

Contents

These supplemental materials include descriptions of:

1. the model used in the Monte Carlo simulations
2. the Mössbauer spectroscopy under magnetic field results and analysis
3. the temperature-dependent ZFC, FC, TRM, and IRM measurements
4. the determination of the freezing temperature in ac-susceptibility measurements
5. the magnetic-field dependent DCD and IRM magnetization measurements
6. the different thermal protocols used in the memory experiments.
7. Monte Carlo simulations of the memory plots considering solely intraparticle interactions

1. The model

The model has been described in detail in reference [1]. We consider a dense assembly of N spherical ferrimagnetic nanoparticles with core/surface morphology, located randomly on the nodes of a cubic lattice inside a box of 10α x10α x10α where α is the smallest inter-particle distance. Each nanoparticle is described by a set of three classical spin vectors one for the core $\vec{s}_{1i}$ and two for the surface $\vec{s}_{2i}$ and $\vec{s}_{3i}$ with magnetic moment $\vec{m}_{n,i} = m_n \vec{s}_{n,i}$ where $m_n = M_n V_n / M_s V$ i=1,...,N (total number of particles), n=1 stands for the core and n=2,3 for the "up" and "down" surface sublattices of the nanoparticle, respectively (see Figure S1). V is the particle volume and M_S its saturation magnetization. V_n and M_n are the volume and the saturation magnetization of the core, the "up" and the "down" surface sublattices spins.

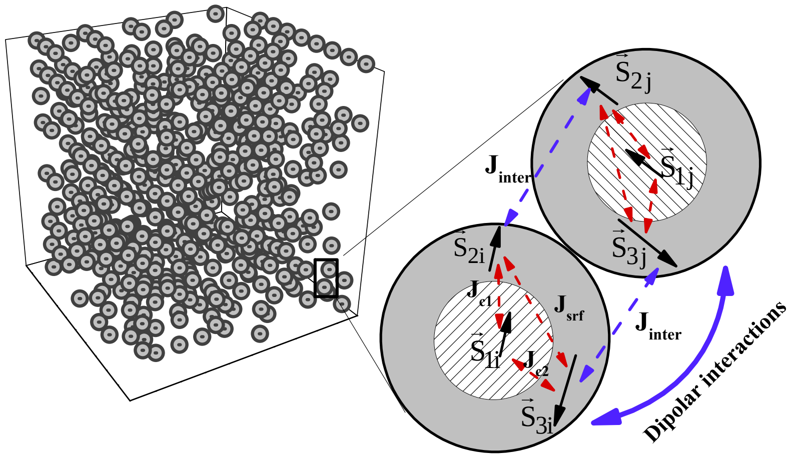

FigureS1 Schematic representation of the nanoparticles assembly (left) and a pair of nanoparticles in contact(right). In the right panel the red arrows represent the intra-particle exchange interactions in each nanoparticle with core (1 spin) / surface (2 spins) morphology and the blue arrows represent inter-particle dipolar (full line) and exchange (broken line) interactions.

The total energy of the system for the N nanoparticles is [1]:

$$
\begin{aligned}
E = & -\frac{1}{2}\sum_{i=1}^{N}\left[J_{c1}\left(\vec{s}_{1i}\cdot\vec{s}_{2i}\right)+J_{c2}\left(\vec{s}_{1i}\cdot\vec{s}_{3i}\right)+J_{srf}\left(\vec{s}_{2i}\cdot\vec{s}_{3i}\right)\right] \\
& -\sum_{i=1}^{N}K_c V_1\left(\vec{s}_{1i}\cdot\hat{e}_{1i}\right)^2-\sum_{i=1}^{N}K_{srf}\left[V_2\left(\vec{s}_{2i}\cdot\hat{e}_{2i}\right)^2+V_3\left(\vec{s}_{3i}\cdot\hat{e}_{3i}\right)^2\right] \\
& -\frac{1}{2}g\sum_{\substack{i,j=1\\ i\neq j}}^{N}\left(\sum_{n=1}^{3}m_{ni}\cdot\vec{s}_{ni}\right)D_{ij}\left(\sum_{n=1}^{3}m_{nj}\cdot\vec{s}_{nj}\right) \\
& -\frac{1}{2}J_{inter}\sum_{<i,j>}\left[\left(\vec{s}_{2i}\cdot\vec{s}_{3j}\right)+\left(\vec{s}_{3i}\cdot\vec{s}_{2j}\right)\right]-\sum_{i=1}^{N}\sum_{n=1}^{3}Hm_{ni}\left(\vec{s}_{ni}\cdot\hat{e}_{h}\right)
\end{aligned}
\qquad \text{(S1)}
$$

The first, second and third energy term describe the Heisenberg exchange interaction between the core spin and the two surface spins (interface coupling J_{c1} and J_{c2}), and the exchange interaction between the surface spins (surface coupling J_{srf}), respectively of all nanoparticles. The fourth and the fifth terms give the anisotropy energy for the particles' core (K_C) and the particles' surface (K_{srf}) ($\hat{e}_i$ being the anisotropy easy-axis direction). Each spin has a uniaxial easy anisotropy axis randomly oriented. The sixth term gives the dipolar interactions between nanoparticles, where D_{ij} is the dipolar interaction tensor [1]. The next term describes the inter-particle Heisenberg exchange interactions (J_{inter}) where <i,j> denotes summation over nearest neighbors. The last term is the Zeeman energy ($\hat{e}_h$ being the direction of the magnetic field).

The energy parameters, as they are inserted in the simulations, have been normalized by the factor $20 \times K_C V_1$, where V_1 is the core volume of the nanoparticle, so they are dimensionless. The description of the model parameters are given in details in reference [1]. The mesoscopic magnetic moments are m_1=0.1, m_2=0.5 and m_3=0.4. The reduced parameters are the intra-particle exchange-coupling constants among the core spin and the surface spins j_{c1} = 0.5, j_{c2} = 0.45, j_{srf} = −1.0; the anisotropy constants for the core is k_C = 0.05 and for the surface is k_{srf} = 1.0; the interparticle exchange-coupling constant is j_{inter} = −0.50 and the dipolar strength is g=3.

The magnetic configuration was obtained by a Monte Carlo simulation, using the standard Metropolis algorithm[2]. For every field and temperature value, the first 500 steps per spin are used for equilibration and the subsequent 5000 steps per spin for thermal averaging. The Monte Carlo simulations results for a given temperature and applied field were averaged over 60 samples with various spin configurations, realizations of the easy-axes distribution and different spatial configurations for the nanoparticles.

Recently there have been several studies on spin glass systems that demonstrate the importance of the system size on the simulations [3,4] of 2D and 3D systems. The Ewald summation has been proven to be an important tool for the treatment of the long range dipolar interactions and the study of the dynamical effects in systems of nanoparticles. In our study, for the dipolar energy calculation, the Ewald summation technique has been implemented using periodic boundaries in all directions. For the memory effects we have used the protocol described in the main text (consistent with the experimental one).

2. Mössbauer spectrometry under intense external magnetic field

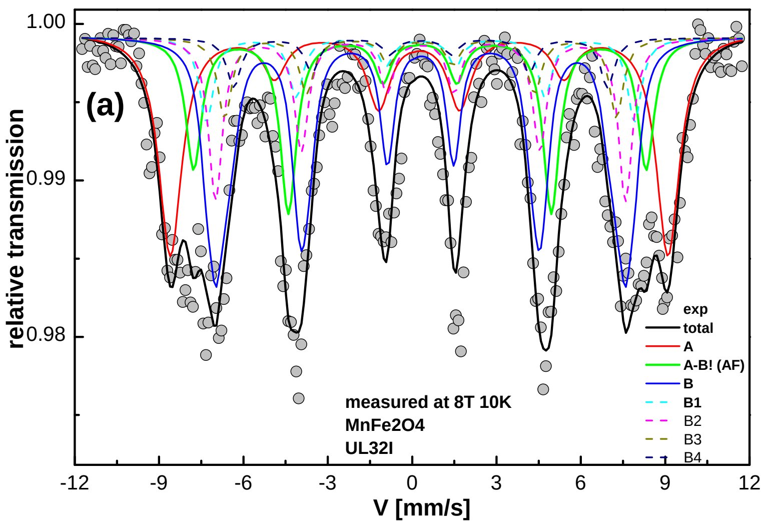

Figure S2: ^{57}Fe Mössbauer spectrum measured at 10 K under high magnetic field (8 T) and fitted with 3 principal components. Sub-spectra correspond to A-site, AF-like component and B-site (fitted using a Lorentzian distribution (dashed lines)).

^{57}Fe Mössbauer spectra were recorded using a ^{57}Co/Rh γ-ray source mounted on an electromagnetic transducer with a triangular velocity form. The samples consist of a thin layer of about 40 mg of the powdered compound located in a sample holder. The spectra were obtained at 10 K in an 8 T (B_{app}) field oriented parallel to the γ-beam and in the range 4- 65 K in zero field; all the spectra were analyzed using the program Mosfit. The hyperfine structure was modeled by a least-square fitting procedure involving Zeeman sextets composed of Lorentzian lines. To describe the broadening of lines, several magnetic subcomponents have been considered where the isomer shift (δ), quadrupolar shift (2ε), linewidth and effective field (B_{eff}) values were free during the refinement as well as the intensities of intermediate lines

(2,5) resulting from the angle between the hyperfine field and the γ-beam, while the ratio of absorption areas of the external/internal lines is systematically equal to 3. The isomer shift (IS) values were referred to those of α-Fe at 300 K. The recorded spectrum (figure S2) consists of a pure magnetic sextets typical of a blocked magnetic states. The hyperfine structure was modelled by a least-squares fitting procedure involving Zeeman sextets composed of Lorentzian lines. The recorded spectrum has a complex shape and it is broadened while the intermediate lines show somehow high intensity. The broadening of the B-sites lines are more pronounced than the A-sites lines. We don't arrive to reproduce, by computer fit, correctly the experimental spectrum using a model with two sites A and B as usually expected in the case of spinel ferrite structure (weakly canted ferrimagnetic structure). We should note that, regardless the fit, we found always one component with big canting angle $\beta \cong 90°$ (angle between hyperfine field and applied field like AF behavior). To reproduce this spectrum, we propose a model which consist with 3 principal components (3 sites), A, B and AF one. The broadening of the hyperfine structure of B-sites is more pronounced than that of A-site. This significant broadening in B-site is interpreted as due to a distribution of Mn and Fe nearest A-site neighbours. In fact the hyperfine fields at B-site are function of the occupation the six nearest A-sites (Fe and Mn). The sub-spectra of B-site were fitted by mean discrete distribution of magnetic sextets composed of lorentzian lines with different values of hyperfine field. The change on the value of hyperfine field is consistent with the probability that an Fe B-site ion has different nearest-neighbor Mn A-site ions.

	$<\delta>$(±0.01)	2ε	B_{eff}	B_{hf} (±0.5)	θ(±10°)	%(±5%)
**A**	0.43	0.0	54.7	47.9	30	33
**(A – B) AF**	0.46	0.0	49.6	50.2	90	23
**B1**	0.48	0.0	47	51.9	56	10
**B2**	0.48	0.0	45	49.9	56	19
**B3**	0.48	0.0	43	47.9	56	9
**B4**	0.48	0.0	41	46	56	6
**<B>**	0.48	0.0	44.5	49.8	56	

Table S1 : Summary of obtained values of hyperfine parameters - Isomer Shift (δ), Quadrupolar Shift (2ε), Effective field (B_{eff}), Hyperfine field (B_{hf}), Angle (θ), and weight (%) - obtained at 10 K under an external magnetic field of 8T.

As shown Table S1, when the number of Mn nearest neighbors increases, the hyperfine field decreases. In this computer fit, the isomer shift (δ) and angle (β) canting were constrained to be the constant for the different sub-spectrum (we used mean value). The solid line is the envelope of the four sub-spectra. The second component consists of the A-Site, the computer fit is shown in figure S1. The mean values of hyperfine parameters (given in Table S1) are closely to those of A-site. The third site corresponds to an AF component (β ≅ 90°) as shown in figure S2 and Table S1 (this parameter (β) was free during the fitting). This AF component, may be came from site B at surface, as δ and H_{hyp} close enough to those of B site. We can note that the proposed model describes very well the details of the Mössbauer spectrum and that this description allows a good fit to be achieved, as illustrated in Figure S2.

3. Temperature-dependent ZFC, FC, TRM, and IRM measurements

The zero-field cooled (ZFC) magnetization was collected as a function of the temperature on reheating in a small magnetic field after cooling the system in zero magnetic field. The field-cooled (FC) one was collected subsequently after recooling the system in the same magnetic field. After a second recooling in the applied magnetic field, the magnetic field was switched off and the thermo-remanent (TRM) magnetization collected in zero applied magnetic field on reheating. In the case of the isothermal remanent (IRM) magnetization measurements, the system was cooled and the magnetization measured on reheating in zero magnetic field in both cases; yet including halts at constant temperatures during cooling (see section 5 below).

4. Analysis of the ac-susceptibility data

Figure S3 shows the temperature dependence of χ' and χ" both normalized by χ'(f = 1.7 Hz, T = 60 K) for a single frequency; the FC magnetization data is also added in the top inset as M/H. We choose T_f as the temperature for which χ'(T_f,f) deviates from the FC susceptibility by 1% (marked by a dashed vertical line) . Using this criterion, T_f marks the temperature onset of out-of-phase component χ", as suggested by the dashed vertical line at T_f.

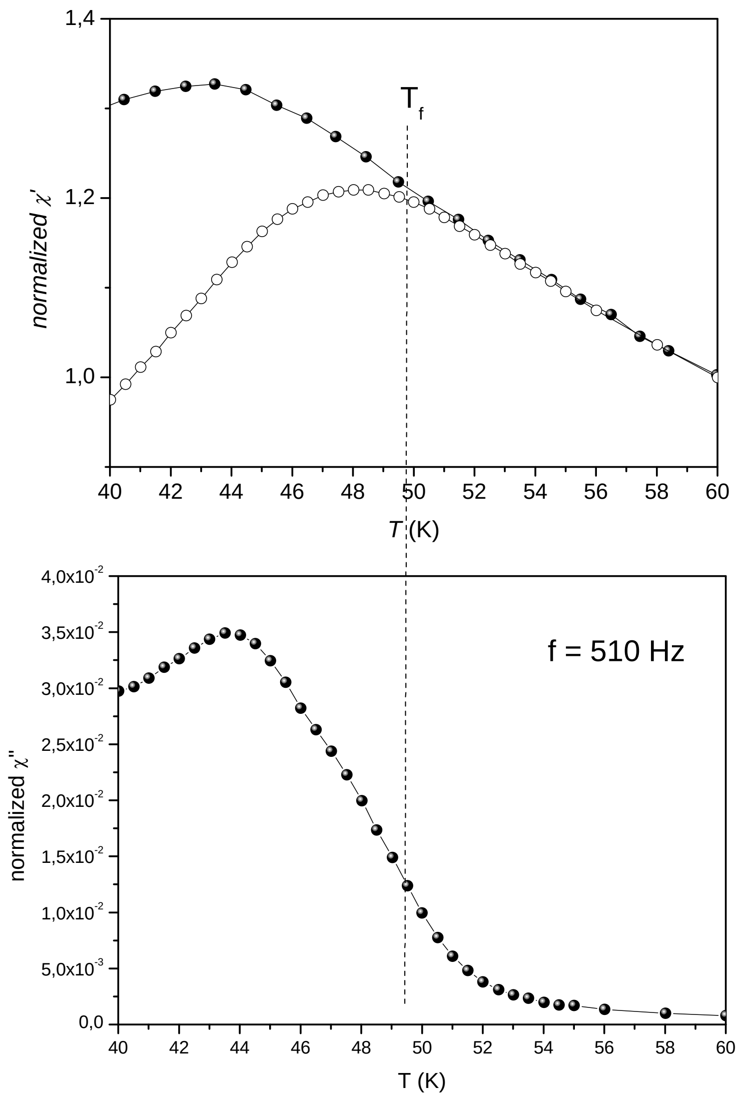

Figure S3: Illustration of the determination of T_f in ac-susceptibility data. Temperature dependence of the In-phase and out-of-phase components of the ac-susceptibility for a given frequency f.

5. Magnetic interparticle interactions: Magnetic-field dependent DCD and IRM magnetization

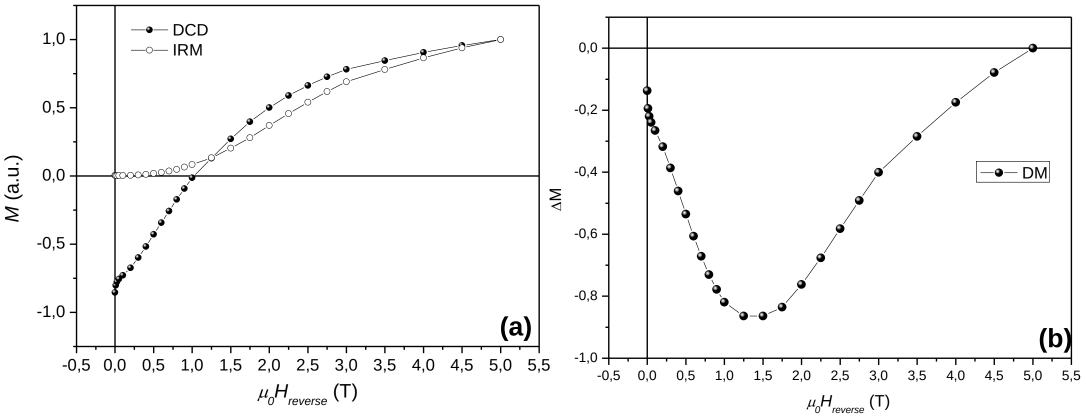

Figure S4: *IRM (open circles) and DCD (filled circles) curves recorded at 5K; (b) ΔM-plot obtained from DCD and IRM curves (equation (2)).*

For deep investigation of interparticle interactions, the magnetic field dependence of remanent magnetization at 5K by IRM and DCD protocol have been investigated The initial state for an IRM measurement is a totally demagnetized sample cooled in zero magnetic field. In the present case, an external field was applied for 10 s; then, it was switched off and the remanence was measured (M_{IRM}). The process was repeated, increasing the field up to saturation. In a DCD measurement, the initial state is the magnetically saturated one. An external field of −5 T was applied for 10 s; then, a small external field in the direction opposite to magnetization was applied and, after 10 s, it was switched off and the remanent magnetization (M_{DCD}) was measured. This was repeated while increasing the field up to +5T

ΔM-plot helps to clarify the interparticle interaction picture. For a system with uniaxial anisotropy and without interparticle interactions, the same energy barrier is calculated from IRM and DCD curves, as in Wohlfarth relation [5]:

$$M_{DCD}(H) = 1 - 2M_{IRM} \qquad (1)$$

Kelly et al. rewrote this expression as [6]:

$$\Delta M = M_{DCD}(H) - 1 + 2M_{IRM} \qquad (2)$$

Negative deviations in *ΔM* are usually taken as indicative of the presence of interactions those stabilize the demagnetized state (i.e., dipole-dipole interactions). Positive values are attributed to interactions promoting the magnetized state (i.e., exchange interactions). In the sample under investigation a clear prevalence of dipolar interactions is observed.

6. Measurements protocols to record TRM and IRM magnetization.

Figure S5 shows the schematic evolution of the temperature (top) and magnetic field (bottom) as a function of time. Thin lines refer to cooling or waiting at constant temperature, while thick lines indicate measurements on re-heating. For example, in a reference TRM measurement (red curve), the system is rapidly cooled down in a magnetic field h from T_{ref} above T_g down to the lowest temperature T_{min}. h is switched to zero and the remanent magnetization is collected on reheating. In a TRM experiment with field stop (FS, blue TRM FS curve), a halt is made at a temperature T_h below T_g during the time t_h, keeping the field to its h value. The halt may be performed for the same duration t_h, albeit after a certain wait time t_w (black TRM FS curve). In TRM ZFS measurements, the magnetic field is switched to zero during the halt instead of being kept as in FS measurements. In IRM ones, the magnetic field is switched to h during the halt and zero otherwise.

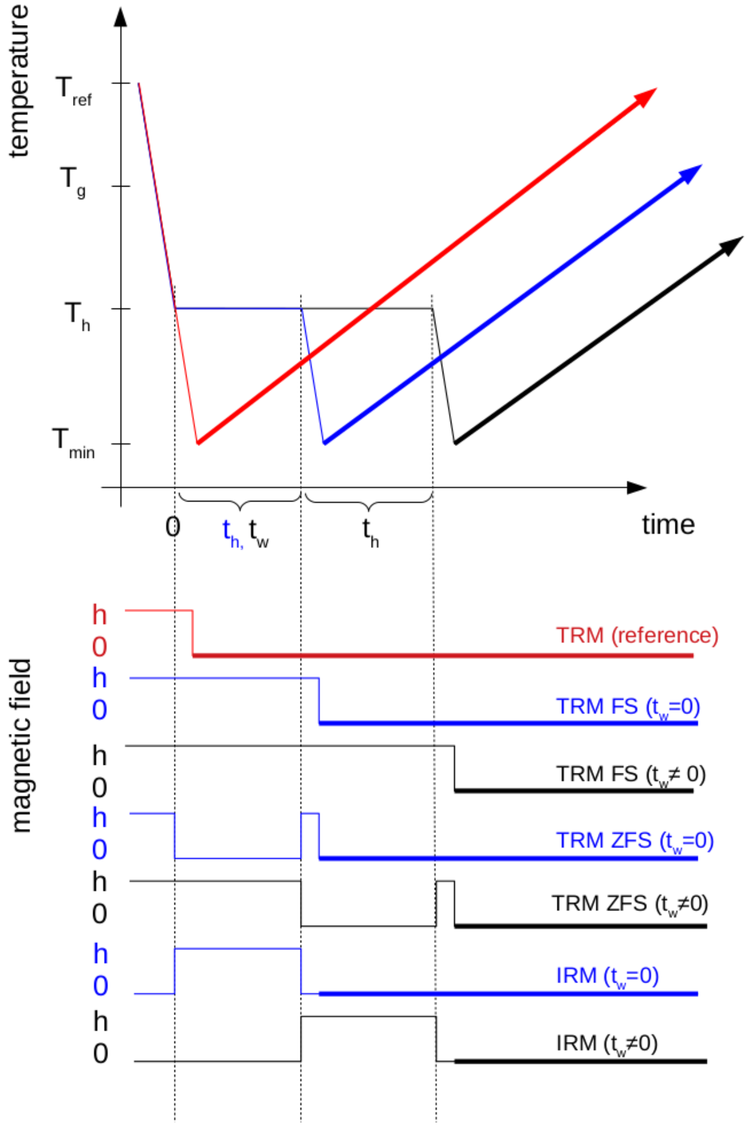

Figure S5: Schematic illustration of the TRM and IRM measurement protocols.

7. Memory plots vs intraparticle interactions in Monte Carlo simulations

In order to give an estimation of the contribution of the nanoparticles core/surface morphology (i.e., intraparticle interactions) in the memory behavior of the system, we have switched off both the dipolar (g=0) and exchange (j_{inter}=0) interparticle interactions. A finite difference ΔM is observed, however much smaller than that obtained for the full system, and shifted to lower temperatures. In that case, the M-T curves of the system are also affected (see Fig. 3d in ref. [1]), as the system turns into a superparamagnet. More results obtained for e.g. different waiting times are necessary in order to fully understand the nature and origin of the ΔM in this case.

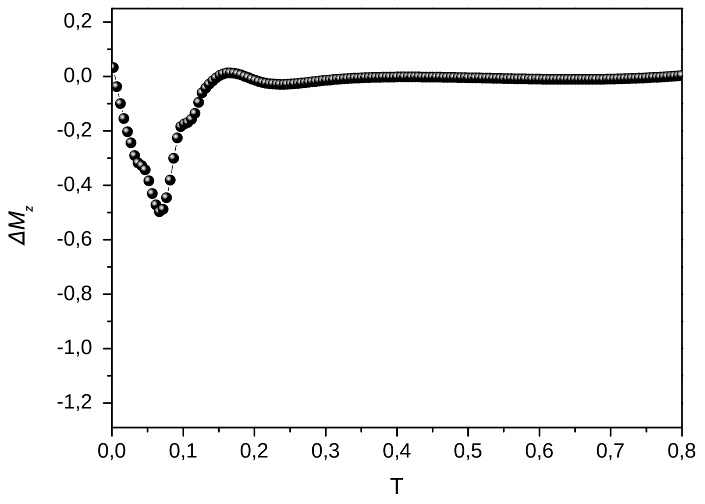

Figure S6: Difference ΔM between ZFC reference and ZFC memory curves for the system where both the dipolar (g=0) and exchange (j_{inter}=0) interparticle interactions are switched off.

References

[1] M. Vasilakaki, G. Margaris, D. Peddis, R. Mathieu, N. Yaacoub, D. Fiorani, et al., *Monte Carlo study of the superspin glass behavior of interacting ultrasmall ferrimagnetic nanoparticles*, Phys. Rev. B. **97**, 094413 (2018); see also G. Margaris, K.N. Trohidou, J. Nogués, Mesoscopic model for the simulation of large arrays of Bi-magnetic core/shell nanoparticles, Adv. Mater. **24**, 4331–4336 (2012).

[2] K. Binder, A.P. Young, Spin glasses: Experimental facts, theoretical concepts, and open questions, Rev. Mod. Phys. **58**, 801 (1986).

[3] T. Nakamura, *Strategy of Solving difficulties in Spin Glass simulations,* Phys. Rev. E **99**, 023301 (2019).

[4] B. McNaughton , M. V. Miloševic, A. Perali , S. Pilati, *Boosting Monte Carlo simulations in spin glasses using autoregressive neural networks,* Phys. Rev E **101**, 053312 (2020) and references therein.

[5] E.P. Wohlfarth, *Relations between Different Modes of Acquisition of the Remanent Magnetization of Ferromagnetic Particles*, J. Appl. Phys. **29**, 595 (1958).

[6] P.E. Kelly, K. O'Grady, P.I. Mayo, R.W. Chantrell, Switching mechanisms in cobalt-phosphorus thin films, IEEE Trans. Magn. **25**, 3881 (1989).
